# Optimization of Friction Welding Process Parameters for 42Cr9Si2 Hollow Head and Sodium Filled Engine Valve and Valve Performance Evaluation

**DOI:** 10.3390/ma12071123

**Published:** 2019-04-05

**Authors:** Fuqiang Lai, Shengguan Qu, Roger Lewis, Tom Slatter, Ge Sun, Tao Zhang, Xiaoqiang Li

**Affiliations:** 1 Guangdong Key Laboratory for Advanced Metallic Materials Processing, School of Mechanical and Automotive Engineering, South China University of Technology, Guangzhou 510640, Guangdong, China; lfq0623@163.com (F.L.); lixq@scut.edu.cn (X.L.); 2 Department of Mechanical Engineering, The University of Sheffield, Mappin Street, Sheffield S1 3JD, UK; roger.lewis@sheffield.ac.uk (R.L.); tom.slatter@sheffield.ac.uk (T.S.); 3 Technical Center, Huaiji Dengyun Auto-parts (Holding) CO., LTD., Huaiji County 526400, Guangdong, China; sunge@huaijivalve.com (G.S.); zhangtao@huaijivalve.com (T.Z.)

**Keywords:** hollow head engine valve, manufacturing method, inertia friction welding, fatigue property, wear property

## Abstract

Due to their design, hollow cavity and filled sodium, hollow head and sodium filled engine valves (HHSVs) have superior performance to traditional solid valves in terms of mass and temperature reduction. This paper presents a new manufacturing method for 42Cr9Si2 steel hollow head and sodium filled valves. An inertia friction welding process parameter optimization was conducted to obtain a suitable process parameter range. The fatigue strength of 42Cr9Si2 steel at elevated temperatures was evaluated by rotating bending fatigue test with material specimens. Performance evaluation tests for real valve components were then carried out using a bespoke bench-top apparatus, as well as a stress evaluation utilizing a finite element method. It was proved that the optimized friction welding parameters of HHSV can achieve good welding quality and performance, and the HHSV specimen successfully survived defined durability tests proving the viability of this new method. The wear resistance of the HHSV specimens was evaluated and the corresponding wear mechanisms were found to be those classically defined in automotive valve wear.

## 1. Introduction

The valvetrain system in an internal combustion engine is used to control the fuel and air exchange and the engine timing. The valves matched with seat inserts in the valvetrain system directly control the gas exchange process [1]. Due to the recent increases in engine performance, combustion chamber temperatures have been rising significantly and improved properties of engine valves are required to accommodate increasingly strict emissions regulations for internal combustion engines. These higher temperatures can often cause premature failure of engine valves. Across engineering, a number of nickel-based superalloys are commonly used for higher-stress and higher-temperature applications such as Inconel 751, Nimonic 80A, but are less commonly used in mainstream automotive products [2]. Valves made of nickel-based superalloys have a relatively higher mass due to their higher density, which would lead to a higher impact force between the contact pair (valve and seat insert). Furthermore, the material cost of engine valves manufactured from nickel-based superalloys is much higher than the valves made of the martensitic or austenitic heat resistant steels. Consequently, effective cooling techniques are also needed to reduce the engine valve temperatures [3].

For many years there have been various attempts to develop cooling techniques for engine valves. Of those, sodium filled valves have often been considered the most promising and they have been widely adopted in recent years. As reported by Heren [4], sodium filled valves were first developed to be applied in piston aircraft engines. The hollow head and sodium filled valve (HHSV) and the hollow stem sodium filled valve are the two main types of hollow engine valves [2]. A HHSV has a relatively larger inner cavity and lower mass than a hollow stem sodium filled valve. In the work reported by Colwell [5], the HHSV had a significantly better temperature reduction effect than the hollow stem sodium filled valve. In addition, a reduction in valve mass is another excellent superiority of HHSV. Based on the research of Baek et al. [6], dynamic characteristics of the valve train system can also be optimized through a reduction of valves mass.

However, the inner chamber filled with sodium in the HHSV makes it more complicated to manufacture. Due to the important role and severe working conditions of the engine valves, HHSVs are required to have excellent performance and reliability. Hence, it is a serious challenge for the engine manufacturers or component suppliers to produce HHSVs through a mass-production technique with high efficiency and the technique must be low cost.

An investigation of HHSVs manufactured from 33Cr23Ni8Mn3N (23-8N) austenitic engine valve steel has been reported in previous work of Lai et al. [7], however, the friction welding process was not detailed. In this paper, investigations of HHSVs manufactured of 42Cr9Si2 martensitic engine valve steel were conducted, especially, the optimization of the friction welding process. Comparative experiments are carried out to study the different effects of the friction welding parameters on the weld quality. Optimized process parameters of 42Cr9Si2 steel were then obtained. The performance evaluation tests utilizing real engine valve and seat insert components were performed in a bespoke bench-top apparatus. The wear resistance of the HHSV specimens was evaluated and the corresponding wear mechanisms are thus presented in this paper.

## 2. HHSV Manufacturing Method

### 2.1. A Review of HHSV Manufacturing Methods

**Figure 1:**
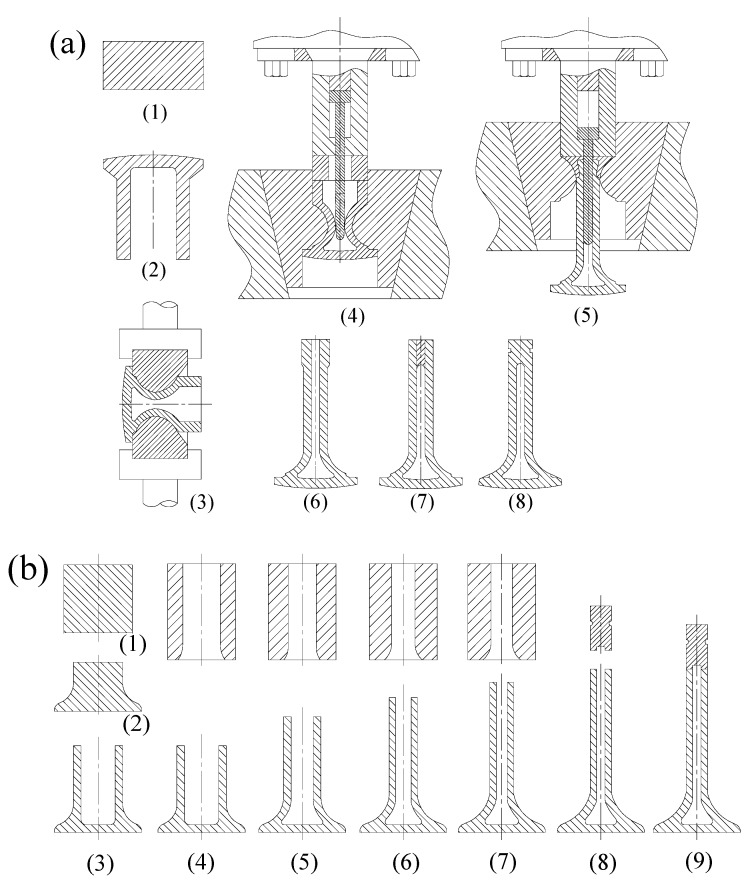
Two methods of hollow head and sodium filled engine valves (HHSV): (**a**) Necking and extruding method proposed by Norton [8]; (**b**) Cold forming necking method proposed by Yoshimura [9].

Different types of manufacturing methods had been invented for HHSVs by many researchers since the Second World War. For instance, Norton [8] proposed a process of making hollow valves, as illustrated in Figure 1a. It has eight main manufacturing steps, as follows: (1) solid bar workblank preparation; (2) preliminary upsetting for initial inner cavity; (3) preliminary necking; (4) extruding of the hollow stem; (5) completion of extruding; (6) upsetting a nubbin end on the valve stem; (7) sodium filling and sealing the hollow stem; (8) completion of machining. Figure 1b presents a cold forming necking method proposed by Yoshimura [9], with nine main manufacturing steps: (1) solid bar workblank preparation; (2) preliminary upsetting; (3) punching for initial inner cavity; (4) first necking; (5) second necking; (6) third necking; (7) completion of necking; (8) sodium filling and shaft end sealing material preparation; (9) sealing the hollow stem and completion of machining. In this method, parts of the valve head are semifinished and then the central lower part of the diameter-increased section is necked down by cold forging performed a number of times to obtain the finished shape.

Furthermore, Beerens et al. [10] developed a type of HHSV with a cylindrical bore produced by electrochemical machining. But it should be noted that the hollow cavity volume of the HHSV can be extended further. In addition, based on the developments in additive manufacturing processes, Additive Layer Manufacturing was applied to design and manufacture hollow engine valves as reported by Cooper et al. [11]. However, Additive Layer Manufacturing is currently too expensive for mass production of engine valves. The material performance produced by Additive Layer Manufacturing should also be further improved to resist fatigue and wear problems at severe work conditions.

### 2.2. The Manufacturing Methods Proposed by HJ Company

**Figure 2:**
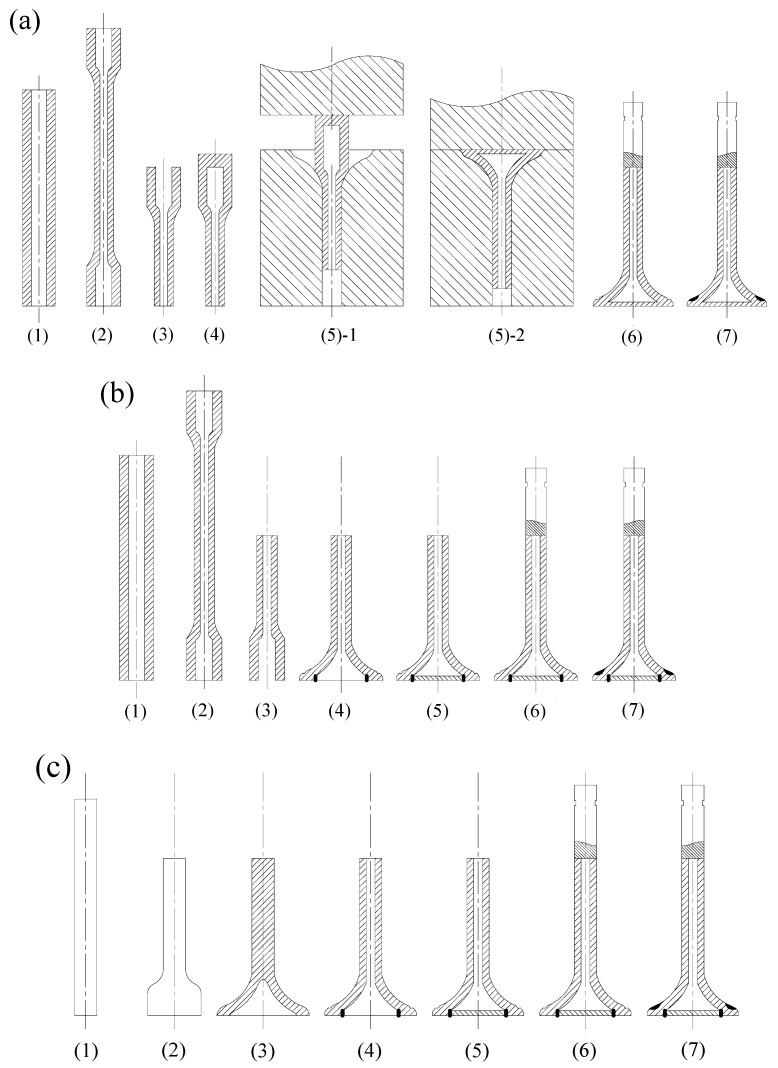
Three methods proposed by Huaiji Dengyun Auto-parts (Holding) CO., LTD. (HJ company): (**a**) hollow engine valve cross wedge rolling and head forging manufacturing method [12]; (**b**) hollow engine valve cross wedge rolling and friction welding manufacturing method [13]; (**c**) hollow engine valve drilling and friction welding manufacturing method [7].

Huaiji Dengyun Auto-parts (Holding) CO., LTD. (HJ company) has invented and proposed three manufacturing methods for HHSVs in recent years, as presented in the two authorized Chinese patents of the HJ company and previous works of Lai et al. [7]. The schematic diagram of the main steps of each method is illustrated in Figure 2.

The first method is referred to as the “hollow engine valve cross wedge rolling and head forging manufacturing method” [12], as presented in Figure 2a. It has seven main processes: (1) preparation of hollow pipe workblank; (2) cross wedge rolling (CWR) on hollow pipe; (3) workblank cutting; (4) sealing at one end bigger hollow stem by friction welding; (5) hollow valve head forging; (6) filling sodium and then sealing at hollow valve stem by friction welding; (7) valve seating face hardfacing and completion of machining. The most difficult stage of this method is the step of hollow valve head forging. Due to the complicated geometry of the workblank and the properties of valve steel materials, the valve hollow head may not always be formed as it is required. There is still a long way to go for this method to be applied in industrial production.

The second method is referred to as the “hollow engine valve cross wedge rolling and friction welding manufacturing method” [13], as presented in Figure 2b. It has seven critical processes: (1) preparation of hollow pipe workblank; (2) cross wedge rolling on hollow pipe; (3) workblank cutting; (4) hollow valve head forming and shaping; (5) sealing at hollow valve head by friction welding; (6) filling sodium and then sealing at hollow valve stem by friction welding; (7) valve seating face hardfacing and completion of machining. As reported by Ji et al. [14], the combination of CWR and forging technique could produce a normal solid engine valve in a highly efficient way. In addition, explorative experiments and numerical analysis for CWR of a 42Cr9Si2 hollow valve were carried out by Ji et al. [15]. The technology maturity of CWR on HHSV should be further developed and improved for better industrial production, especially, the precision forming of inner cavity of hollow stem during CWR processing.

The third method is referred to as the “hollow engine valve drilling and friction welding manufacturing method” [7], as presented in Figure 2c. The method has seven manufacturing processes: (1) preparation of solid bar workblank; (2) upsetting for a bigger solid end; (3) punching on bigger end for hollow valve head; (4) hollow stem valve drilling; (5) sealing at hollow valve head by friction welding; (6) filling sodium and then sealing at hollow valve stem by friction welding; and (7) valve seating face hardfacing and completion of machining.

The advantages and disadvantages of different manufacturing methods for HHSVs are listed in Table 1. As presented in Section 2.1 and Table 1, the HHSVs produced from Additive layer manufacturing method were very expensive, the production of this type of valves is suited only to high value motorsport applications [11]. As for cold forming necking method, only a limited type of valve material can be used, and cold forming prone to cracks. In general, martensitic heat resistant steel, such as 42Cr9Si2 steel, is commonly used for inlet valves. Austenitic alloys and superalloys are used for exhaust engine valves. However, these common materials would be very difficult to use with the cold forming necking method for the production of engine valves, due to the low plasticity of these materials at room temperature. Compared to the CWR process used in the second manufacturing method, the drilling process used in the third manufacturing method has a significantly lower production efficiency. The material utilization rate of the drilling process is lower than the CWR process. However, when the high precision production equipment for the drilling process are prepared, this method is a suitable manufacturing method for hollow stem. The sealing process of the hollow valve head is another critical stage of the second and third method. There are many welding methods to seal the hollow valve head, such as electro-welding and laser beam welding [16]. Based on the exploration and recent manufacturing experience, the third method “hollow engine valve drilling and friction welding manufacturing method” was utilized to produce HHSVs in this work. Low technical difficulty of drilling is needed, and this method could produce the HHSVs in a relatively high production efficiency and low cost, it has been proven to be suitable for mass production of engine valves.

## 3. Friction Welding Process Parameter Optimization

### 3.1. Materials

**Figure 3:**
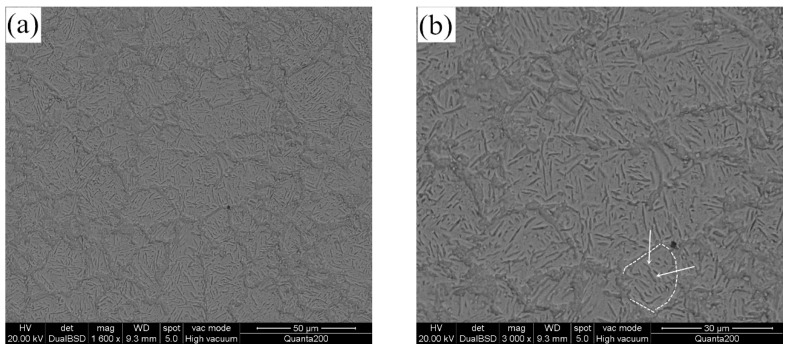
Microstructure of the 42Cr9Si2 steel. (**a**) tempered martensite; (**b**) enlarged view of clearer grain boundaries.

42Cr9Si2 martensitic heat resistant steel is widely used in modern internal combustion engines. The raw steel bars were provided by a commercial steel manufacturer, and the chemical composition of steel was analyzed and confirmed using a spectrograph (Q4 TASMAN, Bruker, Germany). The steel was quenched at 1030 °C for 0.5 h, followed by cooling in oil. Tempering was accomplished at 650 °C for 1.5 h, followed by cooling in air. The steel hardness at room temperature was 37 HRC (Rockwell hardness). The valve seating face hardfacing material was provided by a metal powder company following China National Standard [17]. The chemical composition of seat insert was similarly checked, using a inductively coupled plasma atomic emission spectrometry. The chemical composition of the three materials is presented in Table 2. Figure 3 presents the microstructure of the 42Cr9Si2 steel, the tempered martensite can be observed, as indicated by the white arrows in Figure 3b, and a typical grain boundary is highlighted by a dotted line. The mechanical properties of the 42Cr9Si2 steel at different temperatures were performed by an ultimate tensile testing machine (Instron 8801, Instron Corporation, Norwood, MA, USA), as listed in Table 3. It is found that obvious performance degradation occurs at the highest temperature of 650 °C.

### 3.2. Process Parameter Optimization

#### 3.2.1. Friction Welding Machine and Parameters

**Figure 4:**
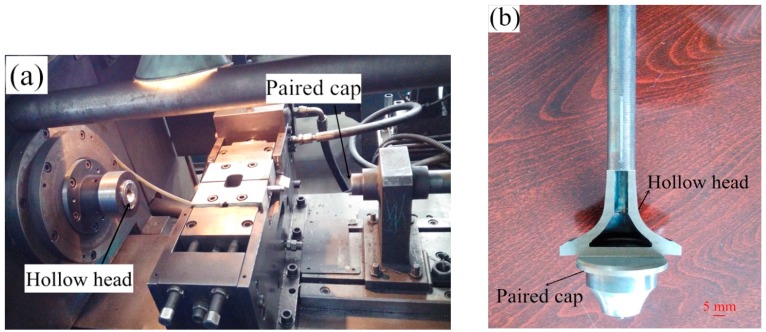
(**a**) Friction welding photo record; (**b**) a typical semi-finished HHSV after welding process.

An important process for manufacturing a HHSV is sealing the hollow valve head through high production efficiency. It has been proved that high quality welds can be produced by friction welding. Friction welding has been applied to join many different types of engineering materials [18]. The advantages of friction welding include: energy saving, low distortion of workpiece and environment protection, as well as no filler metal needed. Friction welding is found to be a suitable way to weld many dissimilar metals. The weld strength is at least as strong or even stronger than the weaker of the two materials being joined [19]. Due to the circular cross-section of a HHSV, rotary inertia friction welding (IFW) was used to seal the hollow head. In this work, the welding was performed in a MTI Model 120B Inertia Friction Welder in the HJ company. A typical progress photo record before the welding process is presented in Figure 4a, and a typical semi-finished HHSV after welding process is presented in Figure 4b.

**Figure 5:**
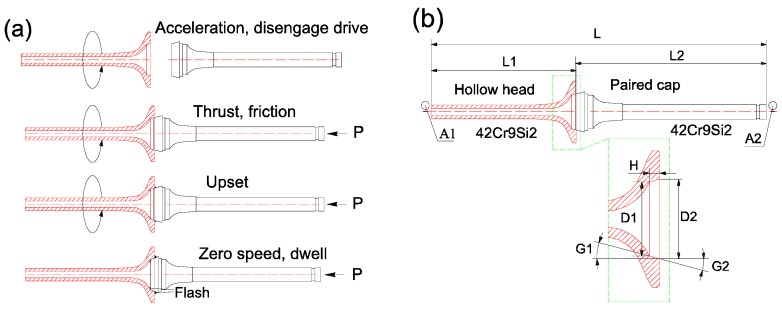
(**a**) Basic steps in two-stage inertia friction welding; (**b**) the geometry of hollow head engine valve workpiece for friction welding.

A two-stage IFW including weld stage and forging (upset) stage was utilized in this work, and its basic steps are illustrated in Figure 5a. Both the hollow head and paired cap workpiece were made of 42Cr9Si2 steel. In the first step, the paired cap workpiece is placed in a stationary chuck, and the hollow head workpiece is installed via a special fixture in a rotating spindle, which supports a flywheel of a specified moment of inertia. The clamped hollow head, spindle and flywheel are accelerated to a specified speed (weld speed) and the drive source is then disconnected. Then, the paired cap is moved, the two workpieces are brought together under a predetermined, constant thrust load (weld pressure). The stored energy of the rotating mass is then released as friction heat. The specimens would be heated locally into a plastic temperature range by the rubbing at the interface. When the spindle speed decreased to a predetermined speed (upset speed), a predetermined load (upset pressure) is applied, causing further axial shortening. The upset pressure is maintained for cooling dwell for some seconds and the weld cycle is completed. This upset would result in the flash presented in the fourth step in Figure 5a. During the friction welding cycle, the contact of interfaces under the pressure lead to atomic diffusion, then, a metallurgical solid state bond would be formed between the interfaces. Based on previous experience of manufacturing engine valves, a conical joint was designed for the friction welding of HHSV, and the detailed geometry of hollow head engine valve workpiece is presented Figure 5b, and the geometry parameters ranges of hollow head valve workpiece are listed in Table 4.

#### 3.2.2. The Relationship Between Weld Quality and Weld Parameters

IFW is a process in which kinetic energy is used to create a fully infused weld joint. As concluded by Olson et al. [20], five factors influence the weld quality: (1) relative velocity of the surfaces; (2) applied pressure; (3) surface temperature; (4) bulk material properties; and (5) surface condition and presence of surface films. It is noted that the first three factors are related to IFW processing parameters, while the last two are related to the properties of the materials being welded. Compared with other welding processes, and due to friction welding disrupting and displacing surface films, the requirement of surface cleanliness is not very strict. The weld surface of HHSV workpieces should still be cleaned and dried before the IFW process to maintain a good and consistent condition, increasing the weld quality. There are five main controllable variables in the two-stage IFW: flywheel mass (I, moment of inertia), weld speed (S_w_), weld pressure (P_w_), upset speed (S_u_), and upset pressure (P_u_). As suggested by Wang et al. [21], in practical applications of rotary friction welding, it should be adjust several welding para meters simultaneously based on the actual condition, such as the welding material properties and the welding machine’s condition. At first, the IFW parameters were gathered empirically, which is also referred to as a “trial and error” method. In order to reduce the processing parameters variable, the flywheel mass was chosen at 0.64 kg·m^2^ according to the “trial and error” method and manufacturing experience, and this parameter was kept the same for all IFW processes in this paper. Table 5 presents the ranges of IFW parameters of HHSV investigated in this paper.

The total kinetic energy available to finish friction welding is determined by flywheel mass and weld speed (initial flywheel speed). The weld pressure is related to the material properties and workpiece contact area. As reported by Olson et al. [20], Equation (1) defines the energy in the flywheel:

(1) $$E=\frac{I\cdot S^{2}}{C}$$

where *E* is energy (J), *I* is flywheel mass (kg·m^2^), *S* is flywheel speed (rpm), *C* = 182.4. For mathematical modelling and parameter calculations, the derived value of “Unit Energy” is defined by Equation (2):

(2) $$E_{u}=\frac{E}{A}$$

where $E_{u}$ is unit energy (J/mm^2^), *A* is faying surface area (mm^2^). The faying surface area *A* can be determined by Equation (3):

(3) $$A=3.1415\cdot H\cdot \cos(G_{1})\cdot \left[\left(D_{1}+H\cdot \tan(G_{1}\right)\right]$$

where *H* is the height of conical faying surface along the direction of rotation axis, *G_1_* is the angle between conical faying surface and rotation axis, *D_1_* is the smaller diameter of conical faying surface. All the geometry parameters are illustrated in Figure 5b. Hence, the unit energy for every IFW parameter of HHSV can be determined by Equation (4). Unit energy can be used to scale data from one size or geometry to another for the same material, and it serve as a first approximation.

(4) $$E_{u}=\frac{I\cdot S^{2}}{C\cdot 3.1415\cdot H\cdot \cos(G_{1})\cdot \left[\left(D_{1}+H\cdot \tan(G_{1}\right)\right]}$$

**Figure 6:**
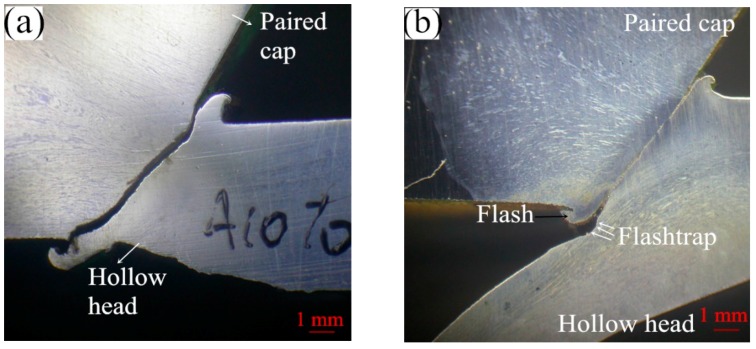
Bad weld quality of HHSV specimens after friction welding process: (**a**) macroscopic crack; (**b**) bad welding metallurgical bonding and bad flash.

Good coaxility between the hollow head and paired cap workpiece ensures good contact conditions of welding interfaces, eliminating the negative effect of misalignment. As presented in Figure 6, the macroscopic crack existed near the welding position, which was caused by the bad coaxility of hollow head and paired cap. Base on the results of the IFW processes of HHSV, the coaxility value between the two workpiece should be lower than 0.12 mm.

After the flywheel mass is determined for the specific workpieces, the weld speed and weld pressure tend to be the two main IFW parameters. When the initial weld speed is at a high level (high total energy), overheating problems in the heat affected zone causes a bad weld quality. However, if the weld speed is too low, the workpieces cannot be welded fully together by the limited friction heat. The temperature distribution in the area of welding is controlled by the weld pressure. As reported by Dawood et al. [22], in the most unpretentious of friction welding, weld pressure on the rotating metal workpiece is escalated to reach an appropriate welding temperature. In order to avoid oxidation, weld pressure must be sufficient to hold the interfaces of workpieces in intimate contact.

**Figure 7:**
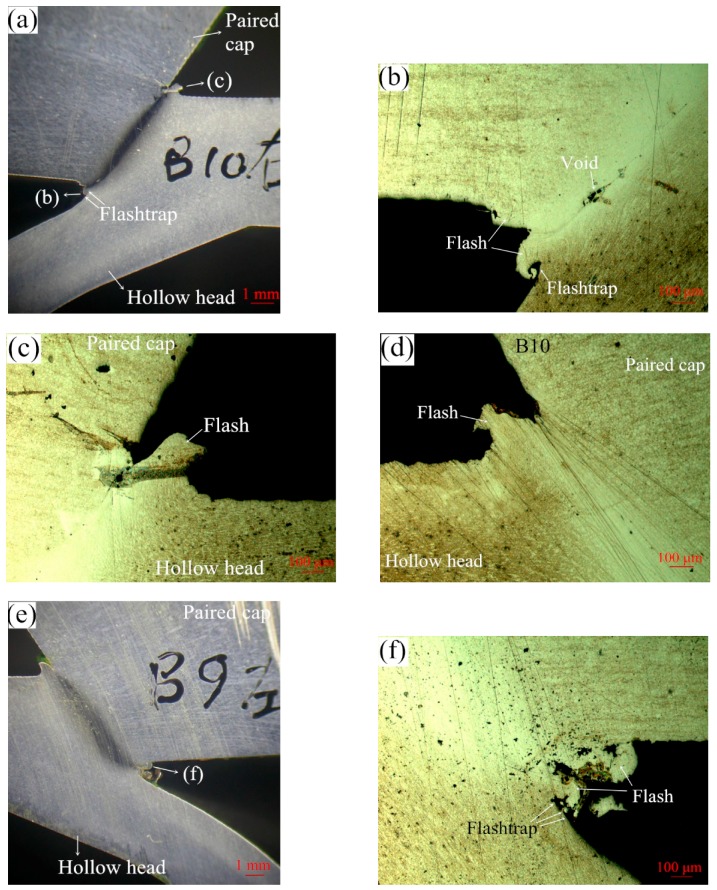
Good weld quality of HHSV specimens after friction welding process: (**a**) cross-section of “B10 right half” HHSV specimen; (**b**) inner edge of friction welding of “B10 right half” HHSV specimen; (**c**) outer edge of friction welding of “B10 right half” HHSV specimen; (**d**) inner edge of friction welding “B10 left half” HHSV specimen; (**e**) cross-section of “B9 left half” HHSV specimen; (**f**) inner edge of friction welding of “B9 left half” HHSV specimen.

**Figure 8:**
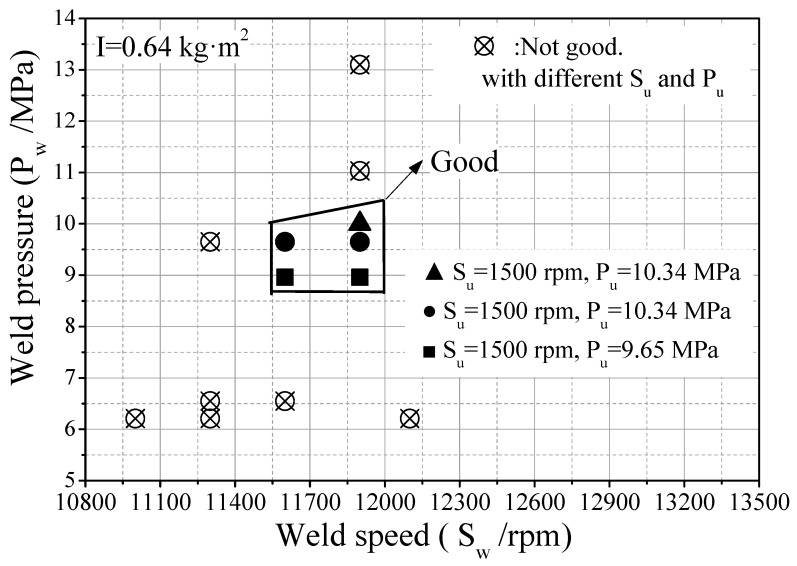
The quality of welding with different friction welding parameters.

If the friction welding parameters are set at suitable levels, the produced flash and weld quality including the welding metallurgical bonding will be good, as presented in Figure 7. In addition, a suitable clearance (flashtrap) should be provided in the inner cavity of the hollow head workpiece to contain the flash produced, as illustrated by white arrows in Figure 7a,b,f. The forging stage begins at a particular critical speed, the amount of forging depends on the energy remaining in the flywheel as well as the upset pressure. Figure 8 presents some results of friction welding experiments of HHSVs, the suitable ranges of friction welding parameters were optimized as follows: weld speed was from 11,600 to 11,900 rpm, weld pressure was from 8.96 to 10.0 MPa; upset speed was 1500 rpm; upset pressure was from 9.65 to 10.34 MPa. Furthermore, according to the calculation of unit energy in Equation (4), a suitable range was determined as being from 1834 J/mm^2^ to 1930 J/mm^2^.

**Figure 9:**
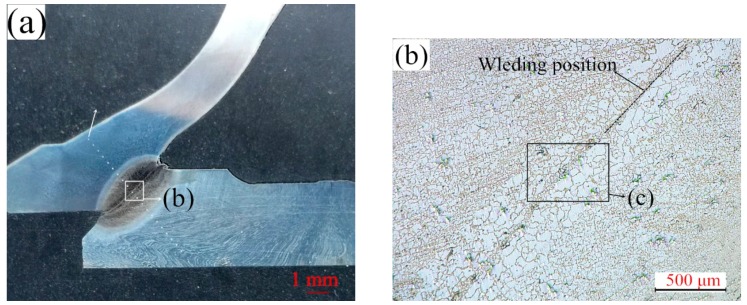

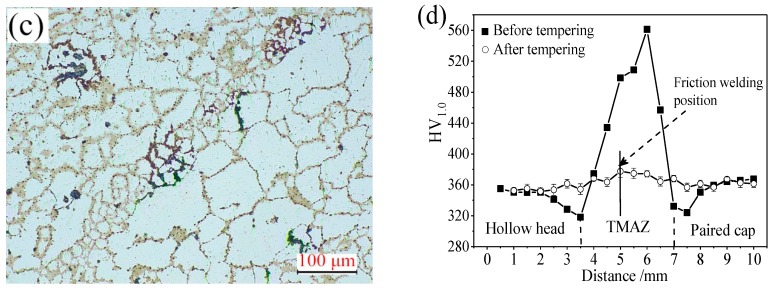
(**a**) Schematic diagram of hardness measurement across thermo-mechanically affected zone (TMAZ); (**b**,**c**) microstructure in TMAZ (**d**) results of hardness revolution across TMAZ before and after tempering.

Due to the severe deformation and quench hardening in the thermo-mechanically affected zone (TMAZ) of friction welding, residual stress was produced and the hardness increased. After IFW processing, heat treatment is required, hence, the HHSV workpieces were subjected to tempering treatment to release the internal residual stress. Figure 9a presents a schematic diagram of the hardness measurement of a sectioned specimen after friction welding, with the white arrow indicating the position where the measurement was started. Good metallurgical bonding existed in TMAZ using the optimized friction welding parameters, as presented in Figure 9b,c. Hardness results across the TMAZ before and after tempering are presented in Figure 9b. It is noted that the hardness in the TMAZ was much higher than the matrix material after friction welding, which can be attributed that material suffering severe plastic deformation and recrystallization.

## 4. Performance Evaluation

### 4.1. Rotating Bending Fatigue Test of Valve Material Specimen

**Figure 10:**
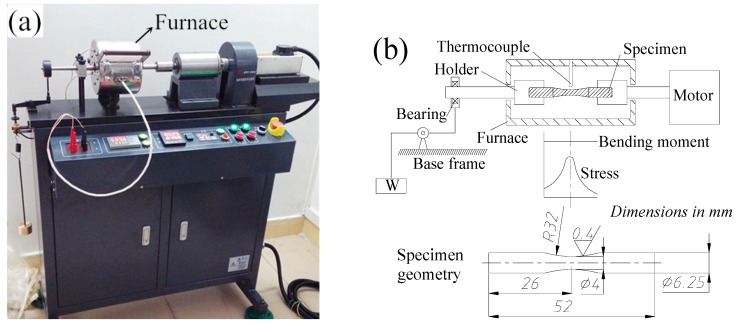
(**a**) Rotating bending fatigue tester; (**b**) schematic diagram of fatigue test and specimen geometry.

Rotating bending fatigue tests for valve material specimens were performed to investigate the fatigue properties of 42Cr9Si2 steel at high temperatures. The fatigue tests were carried out using a cantilever fatigue test apparatus (QBWP, Changchun, China) as illustrated in Figure 10a, and the schematic diagram of fatigue test and specimen geometry is presented in Figure 10b.

The fatigue tests were conducted at a stress ratio of $R=S_{\max}/S_{\min}=-1$ and a frequency of 100 Hz. The temperatures in the furnace were set at 400 °C and 650 °C in the ambient atmosphere, respectively. After ten million cycles had occurred, the fatigue testing was automatically suspended. As indicated by Gere and Timoshenko [23], the inertia forces arising due to a bending load result in bending stress in the cross-sectional of fatigue specimen.

**Figure 11:**
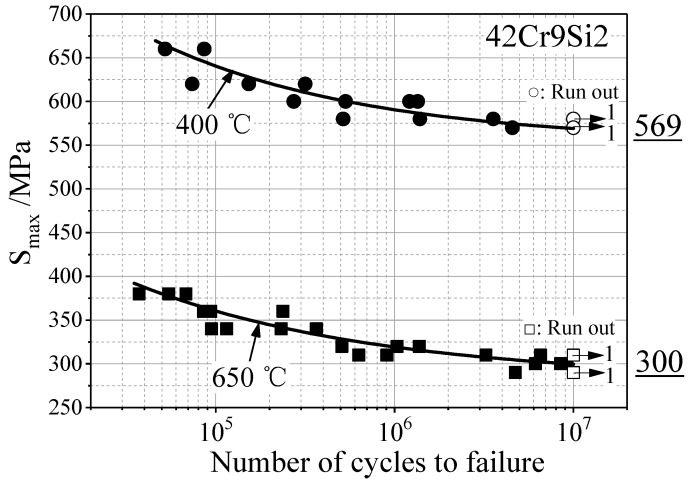
S-N curves of the 42Cr9Si2 steel at the temperatures of 400 °C and 650 °C.

Based on the fatigue results recorded at the two temperatures, the related stress-life curves were produced, as presented in Figure 11. Across engineering, the fatigue strength of material on the base of 10^7^ cycles is often referred to as fatigue limit. It is found that the fatigue limit of 42Cr9Si2 steel was 569 MPa at 400 °C, 300 MPa at 650 °C.

**Figure 12:**
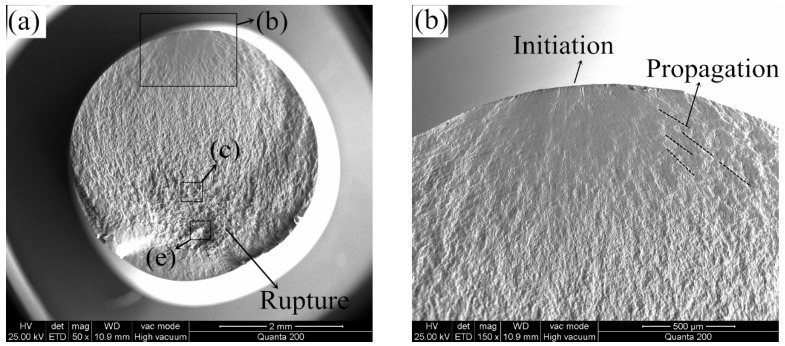

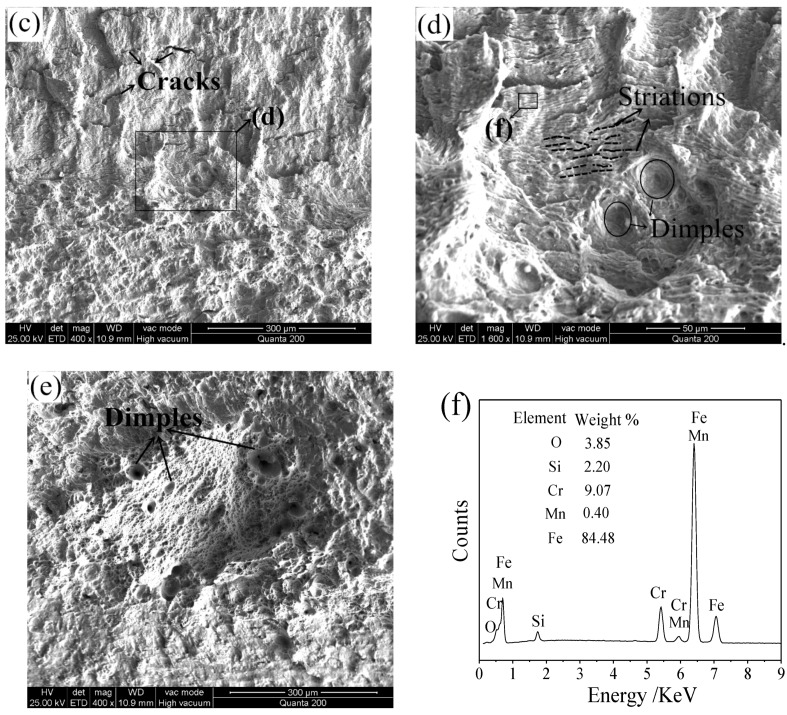
(**a**) Typical fracture surfaces of the specimen ($S_{\max}$ = 320 MPa, N = 1,034,413 cycles) with three regions tested at 650 °C; (**b**) crack initiation region and crack propagation region; (**c**,**d**) near rupture region; (**e**) final rupture region; (**f**) EDS results of corresponding area on fracture surface.

Figure 12 presents the typical fracture surfaces of the specimen tested at 650 °C. As shown in Figure 12a,b, the crack initiation region, the crack propagation region and the final rupture region can be observed on the fracture surface. The initiation position of cracks occurred on the surface of the hourglass shape specimen, due to the maximum bending stress was produced on the surface of specimen during the rotating bending fatigue test. Furthermore, radial streaks can be observed in the crack propagation region. In addition, fatigue striations, cracks and even axial dimples were observed near the rupture region (Figure 12c,d). Numbers of dimples were found in the rupture region (Figure 12e), indicating the fracture type was ductile. According to the EDS results in Figure 12f, a higher oxygen element percentage was detected, it is inferred that the fracture surface was oxidized in the high-temperature test conditions.

### 4.2. Durability Test of Valve Components

**Figure 13:**
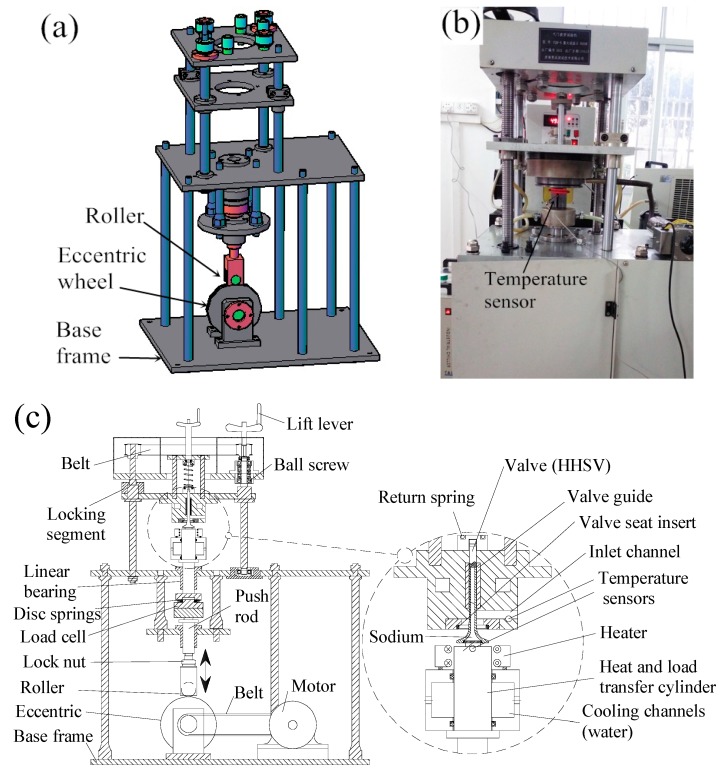
(**a**) Three-dimensional CAD (Computer Aided Design) model of the self-designed bench-top apparatus; (**b**) the manufactured apparatus; (**c**) the schematic diagram of the apparatus.

Utilizing the hollow engine valve drilling and friction welding manufacturing method of HHSV and optimized IFW processing parameters, hundreds of HHSVs made of 42Cr9Si2 steel were produced successfully. Durability tests for real valve components were carried out using a bespoke bench-top apparatus. Figure 13a illustrates the three-dimensional CAD (Computer Aided Design) model of the apparatus. A photo and the schematic diagram of the manufactured apparatus is presented in Figure 13b,c, respectively. The apparatus was driven by a motor and the power was transmitted to the eccentric wheel by a belt. During the durability tests, the determined heat and load can be applied to the central area of the hollow valve head. The apparatus is more thoroughly described in previous work [24].

Three HHSV specimens named A, B and C were tested at different conditions in this work. In addition, the results of two HHSVs made of 33Cr23Ni8Mn3N (23-8N) steel reported in previous research of Lai et al. [7] are given for a comparison. Table 6 presents the properties and configurations of these five HHSVs. Table 7 presents the test conditions and durability test results. The matched seat inserts were selected from a natural gas fuelled diesel engine. Table 2 (Section 3.1) presents the chemical composition of the seat insert. Other test parameters were set as follows: 10 Hz for axial loading frequency, 5 mm for valve lift distance, 0 mm for misalignment of the contact pair (valve and seat insert), and 150 mm/s for valve specimen seating velocity. No lubrication oil and rotation were set between the contact pair during the durability test. The wear scars of contact pair seating faces were measured by a profilometer (MarSurf XC20, Mahr Inc., Esslingen, Germany). The measurements of each specimen were performed at four positions.

**Figure 14:**
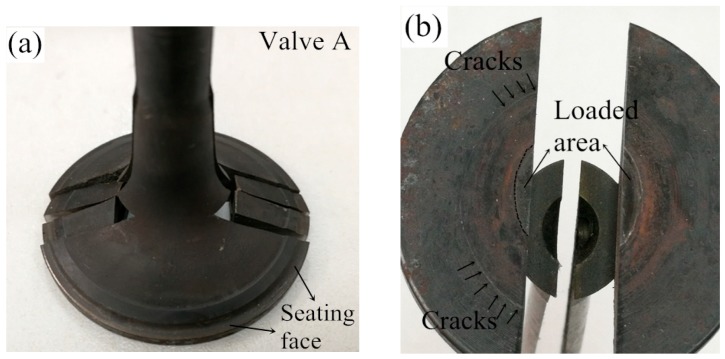
(**a**) Front view of valve A post-test; (**b**) combustion face of valve A post-test.

After the durability test of valve components, the valve specimens were split for inspection. For example, the front view of post-test valve A is presented in Figure 14a. Furthermore, micrographs of the worn surfaces of seating faces and cross-sections obtained from a scanning electron microscope are presented in the later Section 4.4. Test A and B were conducted with a load of 4.2 kN at 650 °C and 400 °C, respectively. Valve A broke after 9300 × 10^3^ impact cycles, and macroscopic cracks were observed on the combustion face of the hollow head (Figure 14b). It should be noted that the cracks were existed out of the loaded area. However, the hollow head of valve B survived after 10^7^ test cycles, no macroscopic and microscopic cracks were observed on the hollow head, which can be attributed to the better material mechanical properties of the valve steel at 400 °C. Furthermore, B and C were tested at 400 °C, but the load in test C was increased to 8.0 kN. Consequently, valve C broke after 2400 × 10^3^ impact cycles with clear cracks on the combustion face of the hollow head, which resulted from the high axial load in the test. In conclusion, HHSVs only survived after the 10^7^ impact cycles at the temperature of 400 °C with a load at 4.2 kN.

### 4.3. Stress Evaluation of HHSV by Finite Element Method

**Figure 15:**
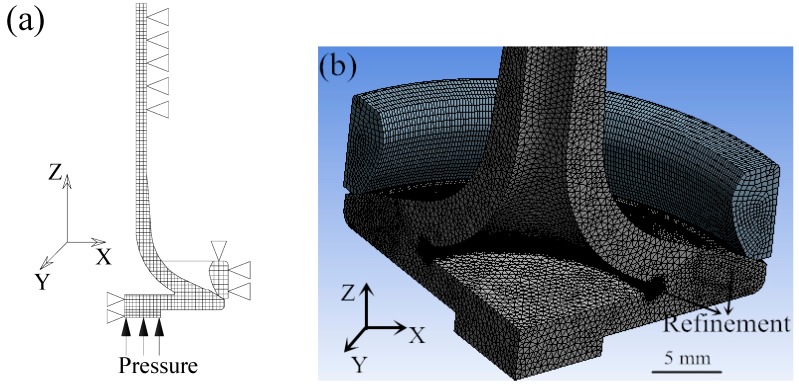
(**a**) Axisymmetric finite element method model of HHSV; (**b**) element mesh of finite element method model of HHSV.

A three dimensional model of the HHSV was firstly established based on its geometry information. Then, mechanical properties of 42Cr9Si2 steel (Table 3 in Section 3.1) at the two temperatures (400 °C and 650 °C) were input to the numerical simulation model, respectively. According to the work conditions and symmetry properties of the HHSV, axisymmetric model of the contact pair was established in the simulation calculation. The circularly nonsymmetric form of the real case resulted from thermal distortion were neglected, as well as the errors in manufacturing and assembly [25]. Hence, a quarter of the HHSV was established in the finite element method model, as presented in Figure 15a. The triangular symbols are used to illustrate the constraints by pointing the constrained direction of the relevant node. For example, because the valve would do the up and down movement in the valve guide, the outer face of valve stem was constrained in the direction of X and Y, and it was not constrained in the direction of Z. Element refinement and element of contact pair were used in the contact region of valve seating face and seat insert, as presented in Figure 15b. ANSYS 14.0 software (ANSYS 14.0, ANSYS Inc., Canonsburg, PA, USA) was used to finish the calculation work.

**Figure 16:**
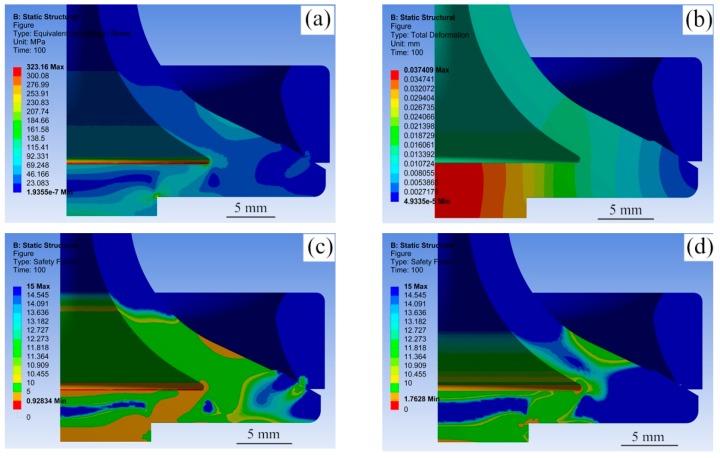
(**a**) Equivalent stress contours of valve A. (**b**) total deformation contours of valve A; (**c**) safety factor contours of valve A; (**d**) safety factor contours of valve B.

Figure 16 presents the typical simulation results of HHSVs. As presented in Figure 16a, the maximum equivalent stress of valve A was 323.2 MPa, and the value of maximum total deformation was 0.037 mm (Figure 16b). The maximum equivalent stress occurred on the junction edge of fillet area and paired cap, indicating these sites would be the position of cracks initiation with a high possibility. The maximum total deformation occurred at the center of hollow valve head. The results of maximum equivalent stress and total deformation of three HHSVs are listed in Table 8. For valve B, at 400 °C, the maximum equivalent stress was 322.8 MPa, and the maximum total deformation was decreased to 0.026 mm, which can be attributed to the higher elasticity modulus of the steel at 400 °C than that at 650 °C. For valve C, when the load was increased to 8.0 kN, the maximum equivalent stress reached 616.3 MPa, and the maximum total deformation was increased to 0.050 mm.

In the area of design of mechanical components, the safety factor $F_{s}$ can be introduced and calculated by Equation (5):

(5) $$F_{s}=\frac{S_{\lim it}}{\sigma_{e}}$$

where $S_{\lim it}$ is the stress limit of material, $\sigma_{e}$ is the equivalent stress. According to the maximum equivalent stress safety tool in the ANSYS software, the material might fail when the maximum equivalent stress exceeds the material limit stress, which means $\sigma_{e}\geq S_{\lim it}$. Consequently, the material could be considered safe when $F_{s}>1$.

It is noted that the frequency of equivalent bending stress in the rotating bending fatigue test was 100 Hz, and 10 Hz was the axial loading frequency in the durability test. According to the research results of a middle carbon steel (bullet train axle steel) from Nonaka et al., the fatigue limit of tests performed at 10 Hz was almost equal to that of tests performed at 400 Hz [26]. Guennec et al. also reported the similar results: a common S–N curve was obtained by normalizing the stress amplitude by the lower yield stress in the usual frequency range of 0.2–140 Hz [27]. Hence, it is reasonable that the fatigue strength obtained from the rotating bending fatigue test can be applied to the stress evaluation.

Consequently, the maximum equivalent stress results were compared to the fatigue strength at ten million cycles of the 42Cr9Si2 steel at high temperatures. For instance, when the stress limit of 42Cr9Si2 steel was set at 300 MPa the fatigue strength of the steel at 650 °C, the safety factor contours determined for valve A are illustrated in Figure 16c. It is noted that the minimum value of $F_{s}$ was 0.924, below the value of 1, indicating the junction edge of fillet area and paired cap was likely to fail after the period of the durability test time. Similarly, safety factor contours of valve B are illustrated in Figure 16d when the stress limit was set at 569 MPa. The minimum value of $F_{s}$ was 1.763, indicating valve B could withstand the test conditions with a long life, and it was verified by the durability test results in Table 6.

### 4.4. Analysis and Discussion

#### 4.4.1. Fatigue Behavior

**Figure 17:**
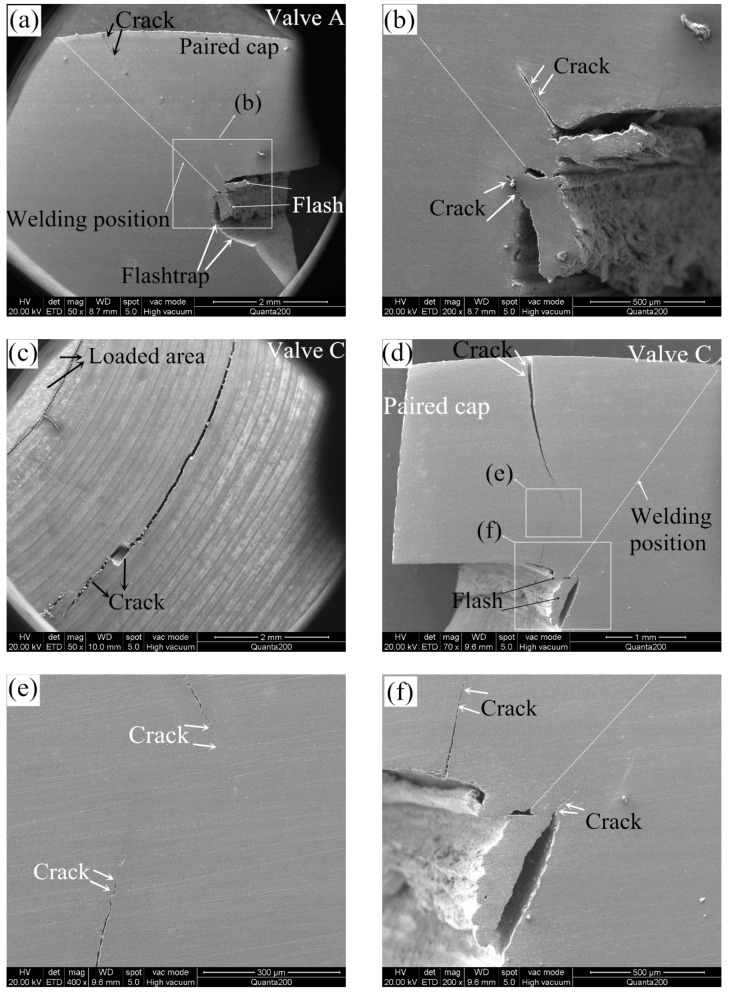
(**a**) Cross-section of valve A after 9300 × 10^3^ test cycles (4.2 kN, 650 °C); (**b**) inner edge of cross-section of valve A; (**c**) combustion face of valve C after 2400 × 10^3^ test cycles (8.0 kN, 400 °C); (**d**) cross-section of valve C; (**e**) enlarged view of cracks on cross-section of valve C; (**f**) inner edge of cross-section of valve C.

Figure 17 presents the cross-sections of hollow head of the two broken HHSVs. As presented in Figure 17a, the outside cracks were observed in the paired cap near the welding position of valve A. During the durability test, the cracks were supposed to be generated by repeated stress. According to the simulation results in Section 4.3, the maximum equivalent stress occurred on the junction edge of fillet area and paired cap, and inner cracks were observed in these area near the welding flash, as presented in Figure 17b. The combustion face of valve C is revealed in Figure 17c, and macroscopic cracks were observed out of the loaded area. Figure 17d,e present the cross-sections of valve C, the position of the cracks in valve C are found to be closer to the center of hollow head, which resulted from the high load. And some inner cracks were also observed near the flash of valve C, as indicated by white arrows in Figure 17f. As mentioned before in Section 3.2.1, the weld strength is at least as strong or even stronger than the weaker of the two materials being joined [19]. In addition, good metallurgical bonding existed in TMAZ using the optimized friction welding parameters, as presented in Figure 7 and Figure 9b,c. It is also found that the position of cracks were between the loaded area and welding position in the paired cap, which was resulted from the load conditions in the durability test. Hence, it can be inferred that the optimized friction welding parameters of HHSV can achieve good welding quality and performance. The cracks in the broken valves after durability test were not initiated in the friction welding position.

#### 4.4.2. Wear Resistance Behavior and Wear Mechanisms

**Figure 18:**
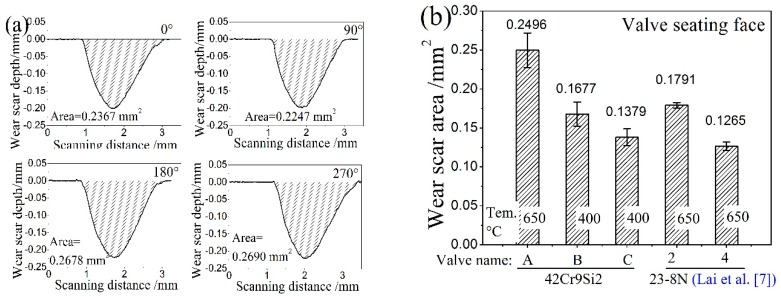
(**a**) Wear scar profiles valve A at four measured positions; (**b**) the comparison of wear scar area of five HHSVs.

Figure 18a presents the wear scar profiles valve A at four measured positions, from which the wear scar area on the seating face can be calculated. The wear scar area of five HHSV specimens is compared in Figure 18b. It is found that the wear loss of valve B tested at 400 °C is significantly lower than valve A tested at 650 °C, which is due to the lower wear resistance of 42Cr9Si2 steel at 650 °C. Friction and wear properties of 42Cr9Si2 steel against 3Cr3Mo3W2V die steel at the temperature of 400 °C and 600 °C were investigated in previous research of Qu et al. [28]. The experimental investigations were conducted using a laboratory pin-on-disc wear tester at dry sliding conditions. Compared to the wear loss of the 42Cr9Si2 steel at 400 °C, the wear loss at 600 °C was obviously increased. Indeed, when the temperature reached 650 °C, the mechanical properties of the 42Cr9Si2 steel decreased further (Table 3), it could be inferred that the wear loss at 650 °C was further increased.

Furthermore, when compared to the valve 2 (10^7^ cycles), the wear loss of valve A (9300 × 10^3^ cycles) is significantly higher, which is due to the lower mechanical properties and wear resistance of 42Cr9Si2 steel than 23-8N steel at 650 °C. Hence, surface enhancement should be applied to improve the wear resistance of the valve seating face. As presented in Figure 18b, compared to valve 2 it had no hardfacing material, and the seating face of valve 4 which was hardfaced with Stellite F alloy had a much better wear resistance [7].

**Figure 19:**
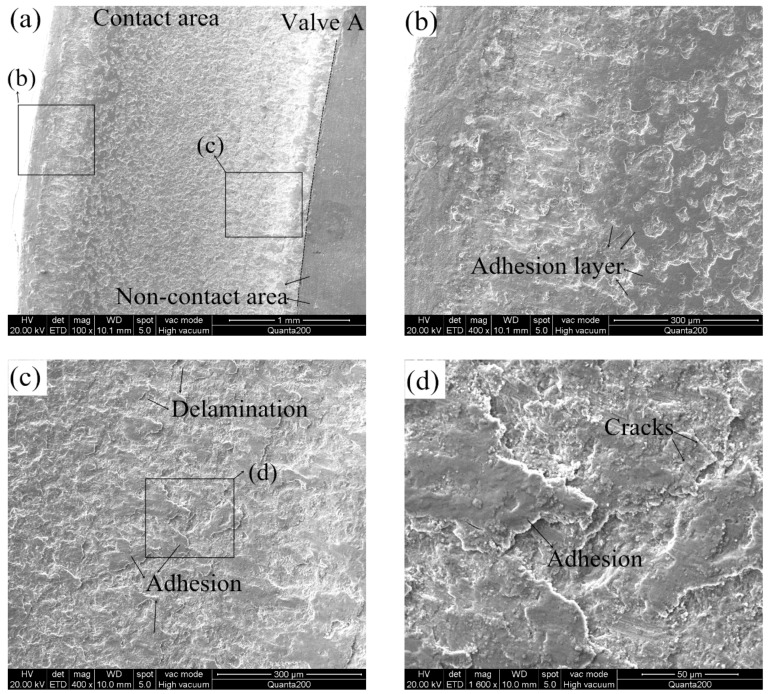

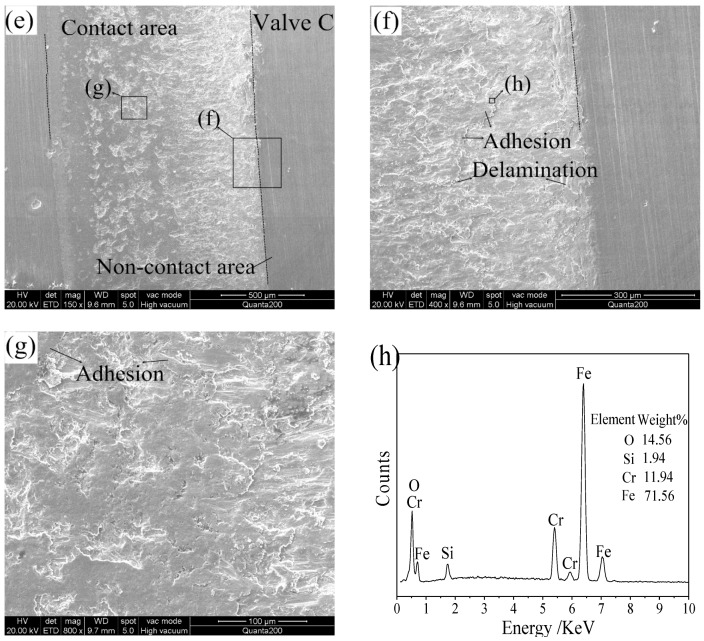
(**a**) Worn seating face of valve A; (**b**) near outer edge of seating face of valve A; (**c**) near inner edge of seating face of valve A; (**d**) enlarged view of adhesion on the worn seating face of valve A; (**e**) worn seating face of valve C; (**f**) near inner edge of seating face of valve C; (**g**) enlarged view of adhesion on the worn seating face of valve C; (**h**) EDS results of corresponding area of valve C.

Lewis and Dwyer-Joyce [29] reported that impact and micro sliding are the two main reasons to describe the wear loss of engine valve contact pairs. The work of quantification of combustion valve sealing interface sliding was reported by Forsberg et al. [30], they presented unique experimental data, acquired using a dedicated technique in a test-rig. The experimental data is complemented by finite element method-simulations. It is found that the sliding length and consequently the wear increase with the increasing combustion pressure. The frictional force on the contact surfaces resulted from the normal force (combustion pressure) when the valve was seated on the seat insert, inducing the surface shear on the interfaces. In addition, lubrication condition, surface roughness, and hardness of the materials of contact surfaces influence the friction factor. The contact conditions of the seating faces in the durability test were more severe than that in an operative engine, leading to a high wear rate. The lower wear rate of the contact pair in an operative engine is attributed to the protective tribofilms can be produced on the sealing surfaces. The importance of oil and particle flow for exhaust valve wear resistance was investigated by Forsberg et al. [31]. It is found that residues from the oils containing additives proved to form protective tribofilms, while the oil without additives promoted agglomeration of wear debris on the sealing surfaces. Furthermore, Forsberg et al. [32] reported that most of the material of the protective tribofilms originated from oil additives, it is also stated that many metallic oxides and other carbon compounds were detected in the combustion system. With the increase of impact cycles at the dry test conditions (no lubrication oil), wear debris was generated from the contact surfaces. Then, debris is impacted and compacted into local tribolayers that cover the surfaces, leading to the formation of contact adhesion. The surface roughness of contact surfaces was then increased. For instance, the adhesion layers were observed on the worn valve seating surfaces, as presented in Figure 19. Simultaneously, the delamination behaviors of the adhesion layers occurred (Figure 19c,f). Hence, adhesive wear is characterized as the predominant wear mechanism of valve and seat insert during the durability test.

The EDS results of the worn area of valve C are revealed in Figure 19h. Compared to the chemical composition of the base material of the 42Cr9Si2 steel (Table 2), the oxygen content on the worn surface was high, indicating that the adhesion layers were oxygen-rich compounds. Consequently, it can be inferred that a kind of oxidative wear is occurring on the valve and seat inserts. As reported by Wang et al. [33], oxidative wear was also identified in the simulation tests of real valve components, the oxide films which formed on the contact surfaces can prevent the direct metal to metal contact, adhesive wear thus then reduced.

Based on the results of rotating bending fatigue tests with material specimens, valve components durability bench-top test results and finite element method stress evaluation results, it can be concluded that the current design (structure and material) and manufacturing (friction welding processing parameters) of the HHSV has passed the durability assessment at 400 °C with a load at 4.2 kN. It is suggested that 42Cr9Si2 steel HHSV could be used as inlet engine valve below 400 °C, but not an exhaust valve at 650 °C. The cracks in the paired cap of broken valves after durability test were not initiated in the friction welding position.

## 5. Conclusions

This paper introduces a new manufacturing method of 42Cr9Si2 steel hollow head and sodium filled valves (HHSVs). The process parameter optimization to successfully achieve inertia friction welding (IFW) of 42Cr9Si2 steel HHSV are also presented. Performance evaluation tests utilizing engine valve components were then conducted in a special-designed bench top tester and the conclusions can be drawn as follows:

(1) The IFW is a suitable manufacturing method for HHSV to seal the hollow head.
(2) The suitable range of IFW parameters of 42Cr9Si2 steel HHSV were optimized as follows:
  unit energy was from 1834 J/mm^2^ to 1930 J/mm^2^,
  weld speed was from 11,600 to 11,900 rpm,
  weld pressure was from 8.96 to 10.0 MPa,
  upset speed was 1500 rpm,
  upset pressure was from 9.65 to 10.34 MPa.
(3) The HHSV survived in test at load of 4.2 kN and test temperature of 400 °C.
(4) 42Cr9Si2 steel HHSV could be used as inlet engine valve below 400 °C, but not an exhaust valve at 650 °C. The cracks in the broken valves after durability test were not initiated in the friction welding position.
(5) The wear mechanisms are classified as adhesive wear and oxidative wear.

## Figures and Tables

**Table 1:** Comparison of different manufacturing methods for HHSVs. CWR: cross wedge rolling.

Methods.	Advantages	Disadvantages
Necking and extruding method [8]	Good mechanical properties of hollow stem after extruding.	Only suitable for large-sized valves.
Cold forming necking method [9]	Manufactured without high temperature, so energy-efficient.	Limited type of valve material Cold forming prone to cracks.
Electrochemical machining [10]	Only one additional process compared to the hollow stem valve.	The hollow cavity volume should be extended further.
Additive layer manufacturing [11]	Manufacturing hollow head valves with any structure and shape.	High production cost, the material performance can be improved.
CWR and head forging [12]	High production efficiency of CWR.	Difficult to achieve precision hollow stem and hollow head.
CWR and friction welding [13]	High production efficiency of CWR.	Difficult to achieve precision hollow stem.
Drilling and friction welding [7]	Low technical difficulty of drilling.	Need precision drilling equipment, low material utilization rate.

**Table 2:** Chemical composition of the 42Cr9Si2 steel and Stellite F and seat insert (wt.%). Bal.: Balance.

Composition	C	Cr	Mn	Si	Ni	Mo	W	Co	Fe
42Cr9Si2	0.42	8.4	0.48	2.38	0.17	–	–	–	Bal.
Stellite F	1.5	24.0	≤0.50	0.9	21.0	≤0.60	11.5	Bal.	≤3.0
	~2.0	~27.0		~1.3	~24.0		~13.0		
Seat insert	0.75	19.0	0.20–	1.75	1.15	–		–	Bal.
	~0.85	~20.5	0.60	~2.25	~1.65				

**Table 3:** Mechanical properties of the 42Cr9Si2 steel.

Temperature, T (°C)	Tensile Strength, s_T_ (MPa)	Yield Strength, s_Y_ (MPa)	Elastic Modulus, E (GPa)	Elongation (%)	Reduction of Area (%)
25	1096 ± 16	901 ± 19	222 ± 18	11 ± 1.6	31 ± 2.5
400	959 ± 13	792 ± 18	196 ± 14	12 ± 1.6	40 ± 1.3
650	512 ± 12	454 ± 15	137 ± 9	19 ± 3.6	57 ± 5.1

**Table 4:** The geometry parameters ranges of hollow head valve workpiece.

Coaxility (mm)	Depth (mm)	Angle (°)	Diameter (mm)
0.08–0.20	2.4–3.6	7–45	26–30.49

**Table 5:** Inertia friction welding processing parameters ranges.

Weld Speed (S_w_/rpm)	Weld Pressure (P_w_/MPa)	Upset Speed (S_u_/rpm)	Upset Pressure (P_u_/MPa)
11,000–12,100	6.21–10.0	1000–5400	7.58–10.34

**Table 6:** Properties and configurations of HHSV specimens.

Valve Name	Head Material	Stem Material	Seating Face Material
valve A	42Cr9Si2	42Cr9Si2, nitriding	42Cr9Si2, nitriding
valve B	42Cr9Si2	42Cr9Si2, nitriding	42Cr9Si2, nitriding
valve C	42Cr9Si2	42Cr9Si2, nitriding	42Cr9Si2, nitriding
valve 2 in Lai et al. [7]	23-8N	23-8N, nitriding	23-8N, nitriding
valve 4 in Lai et al. [7]	23-8N	42Cr9Si2, nitriding	Stellite F

**Table 7:** Durability test conditions and results.

Test	Valve	Seat Insert	Temperature (°C)	Axial Load (kN)	Axial Pressure (MPa)	Cycles (10^3^)	Result
A	valve A	seat insert A	650	4.2	23.78	9300	Broken
B	valve B	seat insert B	400	4.2	23.78	10,000	Run-out
C	valve C	seat insert C	400	8.0	45.30	2400	Broken
2 in Lai et al. [7]	valve 2	seat insert 2	650	4.2	23.78	10,000	Run-out
4 in Lai et al. [7]	valve 4	seat insert 4	650	4.2	23.78	10,000	Run-out

**Table 8:** Finite element method results of three HHSVs.

Test	Temperature (°C)	Load (kN)	Maximum von Mises (MPa)	Maximum Deformation (mm)
A	650	4.2	323.2	0.037
B	400	4.2	322.8	0.026
C	400	8.0	616.3	0.050
